# Concentrating and sequestering biomolecules in condensates: impact on plant biology

**DOI:** 10.1093/jxb/erac497

**Published:** 2022-12-14

**Authors:** Fanourios Mountourakis, Ioannis H Hatzianestis, Stella Stavridou, Peter V Bozhkov, Panagiotis N Moschou

**Affiliations:** Department of Biology, University of Crete, Heraklion, Greece; Institute of Molecular Biology and Biotechnology, Foundation for Research and Technology – Hellas, Heraklion, Greece; Department of Biology, University of Crete, Heraklion, Greece; Institute of Molecular Biology and Biotechnology, Foundation for Research and Technology – Hellas, Heraklion, Greece; Department of Biology, University of Crete, Heraklion, Greece; Department of Molecular Sciences, Uppsala BioCenter, Swedish University of Agricultural Sciences and Linnean Center for Plant Biology, Uppsala, Sweden; Department of Biology, University of Crete, Heraklion, Greece; Institute of Molecular Biology and Biotechnology, Foundation for Research and Technology – Hellas, Heraklion, Greece; Department of Plant Biology, Uppsala BioCenter, Swedish University of Agricultural Sciences and Linnean Center for Plant Biology, Uppsala, Sweden; Bielefeld University, Germany

**Keywords:** Enzyme regulation, phase separation, protein condensation, signaling

**Biomolecules can exist in a variety of forms, ranging from single entities to mesoscale assemblies akin to small organelles, also known as ‘biomolecular condensates’. The formation of biomolecular condensates is expedited by phase separation, in which molecules de-mix to form dilute and condensed phases. Phase separation results in concentrating or sequestering certain molecules, thus altering their abundance or other features in the phases and in this way inhibiting or promoting biochemical reactions. Here, we discuss recent research implicating biomolecular condensates in the regulation of biochemical reactions in plants.**

## Condensation can modulate biochemical pathways

The dissonance in terms of properties between small molecular assemblies with definite stoichiometries and mesoscale assemblies of heterogeneous size, molecular mass, composition, and presumably stochastic stoichiometry prompts a reassessment of basic organizational principles in cells. Despite this stochasticity, mesoscale assemblies output highly selective biochemical reactions underlying cellular- and organismal-level responses at precise locations and times (Pappu, 2020).

Phase separation is likely central to the formation of some mesoscale assemblies; we discuss further its biophysical nature in Box 1. Here, we define phase separation as an abrupt state transition whereby molecules with a propensity to stick to one another will separate from their solvent into distinct assemblies forming quasi-one-dimensional compartments (on linear molecules, e.g. DNA), pseudo-two-dimensional patches (on membranes), or bodies (e.g. in the cytoplasm). We refer to all these mesoscale assemblies, which can form anywhere in the cell, using the umbrella term ‘condensates’. Although condensates can arise through liquid–liquid phase separation, their liquidity can be lost under certain microenvironmental conditions, modifications on their components, or when given enough time to age. Hence condensates can attain less fluid but more gel- or solid-like material properties, the transitions being of the utmost importance for their functionality. Below, we summarize examples highlighting how condensates steer plant physiology by modulating certain biochemical reactions (see also Box 2; Supplementary Table S1).

During development, some of the transcription factors regulating auxin outputs, known as AUXIN RESPONSE FACTORs (ARFs), undergo phase separation (Powers *et al.*, 2019). Arabidopsis has 22 ARFs, with most containing an N-terminal DNA binding domain, a middle part with an intrinsically disordered region (IDR), and a C-terminal domain mediating ARF–ARF and ARF–auxin interactions (Box 1). For a review on ARF functions, we refer the reader to (Cancé *et al.*, 2022). ARF7 and ARF19 are nuclear in cells near the root tip, and thereby involved in the transcription of auxin-responsive genes (Powers *et al.*, 2019). Far from the meristematic cells of the root tip, however, these proteins form cytoplasmic condensates hampering nuclear localization, and in this way mitigating auxin responsiveness. Condensation of the ARFs is mediated by a glutamine-rich prion-like domain (PLD; Box 1) within their IDR, while the C-terminal domain also contributes to the condensation, perhaps by facilitating intermolecular interactions between ARFs. ARF condensation in the cytoplasm allows specific cells to get desensitized to auxin when they need to, e.g. when they function as auxin conduits that channel auxin to other cells. When these cells need to regain responsiveness to auxin (e.g. to initiate lateral roots), the ARF condensates undergo dissolution through an unknown mechanism and re-enter the nucleus.

**Box 1. An introduction to the molecular grammar of phase separation**

The past few years have experienced tremendous progress in the evolution of a molecular grammar that underpins phase separation. Protein and RNA are polymers with attractive groups known as ‘stickers’ that form non-covalent and mainly weak interactions. At certain concentrations that are determined by various factors (e.g. temperature, redox state, pH), interactions are enabled among intra- or intermolecular stickers (e.g. protein 1–protein 2 interaction on the cartoon). When reaching a system-specific threshold concentration (*C*_threshold_), the whole system undergoes a transition into at least two or more phases, a process known as phase separation. Here, the dense phases are often referred to as condensates. Stickers are connected by ‘spacers’ that regulate the density transitions by orienting stickers. The ‘stickiness’ (or multivalency) depends on the attraction between charged residues, dipoles, or aromatic groups that are usually provided by the so-called ‘intrinsically disordered regions’ (IDRs; one class are the prion-like domains noted as PrLD, while both belong to the class of low complexity regions). IDRs lack a defined structure and thus can easily expose their stickers. As a cautionary note, proteins that undergo phase separation (e.g. liquid–liquid phase separation) are usually enriched with IDRs, but many examples show that folded domains or nucleic acids also mediate phase separation (e.g. protein 3 with RNA binding domain (RBD) in the cartoon). Condensates that have formed by liquid–liquid phase separation can display properties of water droplets, such as sphericity, to reduce surface tension, along with increased density and viscosity due to the increased internal concentration. Condensates may also exchange molecules with the surrounding dilute phase. Furthermore, some molecules function as ‘scaffolds’ for the condensation process (much like ‘nucleators’), and likely are recruited first in ‘pre-condensation’ assemblies. Yet, other proteins are recruited to the condensate through multivalent interactions with scaffolds, including RNA binding proteins (RBPs) interacting with RNA molecules. These molecules are called ‘clients’ and may affect condensates by regulating their material properties, e.g. liquid-to-solid transitions or formation of aggregates and filaments at high concentrations. At the top right, we depict the domain organization of ARF7 and ARF19 as examples of phase separating proteins (Powers *et al.*, 2019). For an overview of ARF architectures we refer the reader to Cancé *et al.* (2022). Other ARFs (e.g. ARF5, 6, and 8) have significantly shorter IDRs and PrLDs and likely do not form condensates. PTMs, post-translational modifications.

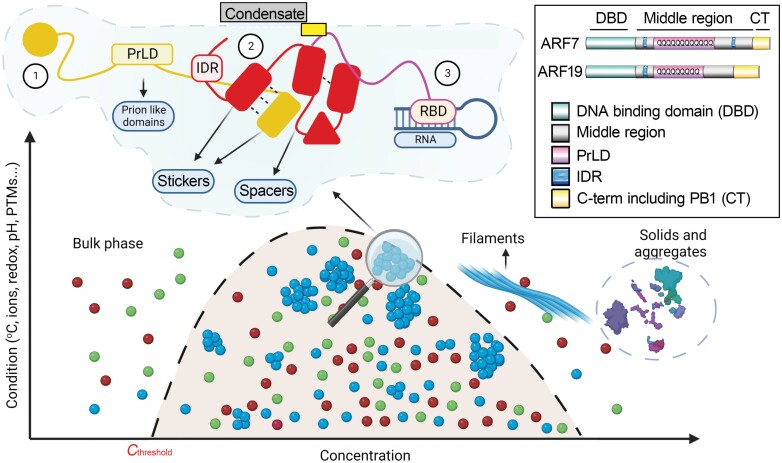

Intriguingly, ARF condensates are not typical non-stoichiometric assemblies as they form a stoichiometric shell (ARF–ARF) at their periphery. This local stoichiometry may play an important role in modulating ARF condensate permeability to additional ARF molecules or other, as yet unidentified, components. In this respect, ARF condensates are reminiscent of proteinaceous conserved bacterial micro-compartments engulfing a cohort of different enzymes to facilitate biochemical reactions (Al-Husini *et al.*, 2018).

Apart from developmental fate decisions, condensates’ reversibility would be perfectly fitted for oscillatory biochemical outputs. EARLY FLOWERING 3 (ELF3) is a transcription factor involved in diurnal rhythms. Increased temperatures induce the formation of ELF3 condensates in the nucleus, a process mediated by the ELF3-PLD (Box 2A, B) (Jung *et al.*, 2020). ELF3 condensates preclude ELF3 molecules from binding their targets on DNA. At low temperatures, however, ELF3 condensates dissolve thereby de-repressing ELF3-dependent transcription of genes inhibiting flowering. It is noteworthy that the ELF3-PLD that mediates condensation displays a variable length and composition among natural populations or species, with longer PLDs richer in glutamate promoting condensation. Hence increased condensation of ELF3 homologs is translated into accelerated flowering in warmer climates. Intriguingly, the yeast Polyadenylate-binding protein (Pab1) is exquisitely sensitive to temperature. A 10 °C increase accelerates Pab1 condensation and likely its activity by ˃300­fold. This increase is much greater than, for example, changes in ion currents for thermosensitive ion channels, which are also tuned for temperature sensing (Sengupta and Garrity, 2013). Therefore, the evolution of condensates as environmental sensors may thus offer a remedy for the non-linear scaling-up of biochemical reaction rates.

## Condensates as catalysts

Recent studies have begun to shed light on the significance of condensates for enhancing enzymatic reactions in plants, which could be achieved through one of the three major mechanisms or their combinations (summarized in Box 2C). In most algae and hornworts, the CO_2_-assimilating enzyme Rubisco condenses within a chloroplast organelle called the pyrenoid as a part of a CO_2_-concentrating process. In *Chlamydomonas reinhardtii*, Rubisco undergoes liquid–liquid phase separation with the IDR-rich protein Essential Pyrenoid Component 1 (EPYC1) to form a pyrenoid (Rosenzweig *et al.*, 2017). Whether co-condensation of Rubisco with EPYC1 lowers *K*_M_ for CO_2_ or increases *k*_cat_ of Rubisco remains unknown.

**Box 2. Examples of condensates and models for enzymatic regulation by condensates**

(A) Selected examples of condensates, along with their localization, and stimuli involved in their formation. Condensates on the cytoskeleton, Golgi, and endoplasmic reticulum have not been identified in plants yet. For condensates not discussed in the text (GDACs, SOSEKI, and photobodies) we provide references in the Supplementary Table S1 summarizing major condensates in plants. (B) Condensates can pause certain pathways by sequestering, for example, transcription factors away from DNA. Example of transcriptional downregulation by ELF3, LUX or ELF4/GI condensates (Jung *et al.*, 2020). See also Supplementary Table S1. (C) Reaction rates in the condensed and dilute phases cannot be equivalent for several reasons. (a) Mass action. Condensates may alter reaction rates by changing the diffusion and local concentration of an enzyme, as well as its substrate(s), product(s), co-factor(s), inhibitor(s), or even competing enzyme(s). Thus, condensates may enhance reactions through increased concentrations of enzymes and/or substrates. This is especially important when the concentrations of the molecules involved are low. (b) Altered environment. The chemical composition of the solvent may change upon condensation creating a microenvironment with altered electrostatics, permeability, and hydrophobicity that will in turn influence substrate binding (*K*_M_). Changes in permeability could in principle restrict the entry of inhibitory molecules into the condensate. (c) Structural switches. Many enzymes are recruited to condensates as scaffolds or clients (see also Box 1 for definitions), through multivalent hetero- or homotypic interactions that may evoke conformational changes affecting substrate *K*_M_. In the example, a scaffold recruits the client enzyme and at the same time undergoes structural modifications enabling enzyme concentration in a confined space, thus enhancing its activity. The aforementioned mechanisms can be used in a combination, further enhancing the specificity and efficiency of single or multi-step reactions (Peeples and Rosen, 2021; Zhang *et al.*, 2021). On the other hand, some enzymatic reactions within condensates or on the interface of two phases might sustain condensate material properties, functionality, and longevity, as exemplified by ATP hydrolysis (Linsenmeier *et al.*, 2022).

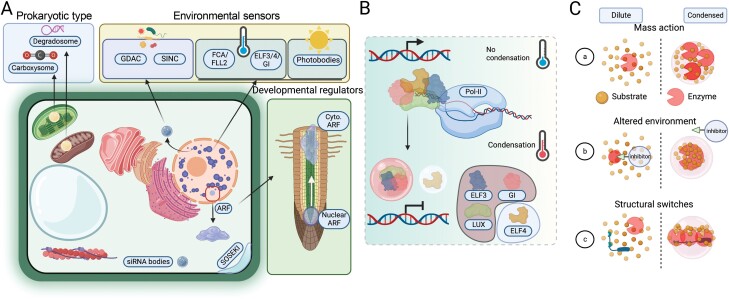

Lying upstream of CO_2_ assimilation in photosynthetic organisms is CO_2_ sensing, which initiates a plethora of signalling pathways central to organismal adaptation to changing CO_2_ concentrations. One potential CO_2_ sensing mechanism shared by fungi and plants is a direct, CO_2_ concentration-dependent activation of a unique group of PP2C phosphatases (Zhang *et al.*, 2022). These enzymes are distinguished by the presence of serine/threonine-enriched IDR, which renders them phase-separating and forming liquid-like, catalytically active condensates in response to elevated CO_2_. Further work is required to demonstrate the physiological relevance of this elegant mechanism coupling enzyme concentration and activation, e.g. in the context of stomatal closure.

The regulation of the Arabidopsis floral repressor *FLC* and other developmental genes relies on the conserved co-transcriptional mechanism of RNA 3ʹ-end processing (*viz.* cleavage and polyadenylation). In the case of *FLC*, 3ʹ-end processing requires the RNA-binding intrinsically disordered protein Flowering Control Locus A (FCA), which forms nuclear condensates in a process dependent on the coiled-coil protein FLL2 (Fang *et al.*, 2019). The FCA bodies are enriched for the enzymatic components of the 3ʹ-end processing. An important next step would be to elucidate whether in FCA bodies the 3ʹ-end processing machinery becomes more active or whether FCA bodies simply serve as a storage depot for the components of this machinery.

The phosphorylation activity of the evolutionarily conserved SNF1/AMPK/SnRK1 protein kinase plays a central role in metabolic response to reduced energy levels under nutritional and environmental stresses. Our recent study revealed a direct association of Arabidopsis SnRK1 catalytic subunit isoforms α1 and α2 with Tudor Staphylococcal Nuclease (TSN) in heat-induced stress granules (SGs) (Gutierrez-Beltran *et al.*, 2021). TSN serves as a scaffold for recruiting many client proteins to SGs, whereas its deficiency represses both SG recruitment and kinase activity of SnRK1α. These data suggest that SG assembly favours SnRK1 activation through an unknown mechanism.

Interestingly, one condensate may promote some and inhibit other biochemical reactions. The condensate formed in response to biotic stress by the NON-EXPRESSOR OF PATHOGENESIS RELATED GENES 1 (NPR1) counteracts effector-triggered immunity (ETI) mediated by salicylic acid (SA) in uninfected cells (Zavaliev *et al.*, 2020). ETI often leads to cellular suicide, which restricts pathogen spread beyond the invasion site. In the adjacent non-infected tissues, systemic acquired resistance prevents extensive damage through NPR1. Three redox-sensitive IDRs punctuated with cysteines in NPR1 upon infection and in the presence of SA mediate NPR1 condensation in the cytoplasm forming the so-called SA-induced NPR1 condensates (SINCs). The SINCs sequester the Cullin 3 RING E3 ligase (CRL3) adaptor promoting its activity, while sequestration of other positive regulators of ETI leads to their inhibition.

## Perspective

We summarize some outstanding questions in Box 3. To further explore and utilize condensates as catalysts of biochemical reactions in plants, we should prioritize the non-invasive measurements of reaction rates in the dense versus the dilute phases in living cells. In the same context, the composition of condensates, their internal organization, the reaction specificities in their different parts, and their regulated dissolution are research directions that need to be further explored. In the long run, these advances together with engineering more efficient phase-separating enzymes, condensate-targeting drugs, or probes, as well as transgenic plants with optimized performance of enzymatically active condensates, will enrich an ­arsenal of tools for artificial manipulation of plant development and resilience. In conclusion, many plant enzymes evolved to occur within or depend on biomolecular condensates, under a selection pressure that likely favored plant adaptation. As a cautionary note, the current model assuming low-affinity unspecific interactions among IDRs to be the major mechanism underpinning biogenesis of biomolecular condensates has been recently criticized (Musacchio, 2022). An alternative model assumes that it is actually site-specific interactions that may be the key drivers of this process.

**Box 3. Outstanding questions**

- How do condensates interact with one another and how could this affect biochemical reactions? In animals, for example, ZNFX1 and WAGO4 transition to perinuclear condensates termed Z granules, immediately adjacent to P granules (Phillips *et al.*, 2012). In adult germline cells, Z granules associate with another type of body, the Mutator foci that contain proteins involved in RNA silencing.
- What are the sequence determinants (including short linear motives) in phase-separating proteins that define such behavior in plants? One route to addressing this question is to examine homologous condensates found in related organisms that live at, for instance, high versus low temperatures when the stimulus for condensation is temperature. For example, a recent study exploited the natural variation in the IDR of FLOE1 condensates in seeds to suggest that FLOE1 condensation propensity can regulate germination timing (Dorone *et al.*, 2021). Such approaches may spur new thinking about the molecular grammar underpinning condensate formation *in vivo*. From agronomical perspective, getting insights into the molecular grammar of condensation could allow the selection or introduction of beneficial traits.
- How is the conformation and activity of an enzyme altered when entering a condensate *in vivo*? It is unclear what the conformation of proteins (and RNAs) that enter a condensed state is. In condensates, proteins may either become protected from degradation or become degraded more; this has also been suggested for RNA molecules (Poudyal *et al.*, 2021). A less folded state may allow stickers to explore their surrounding space and find favorable partner stickers, thereby mediating condensation propensity. The increased rigidity in a folded state could conflict with the need for stickers to explore their surroundings and is, therefore, kinetically disadvantageous (Day *et al.*, 2021). *In vivo* evidence in support of results of increased activity in condensates heretofore obtained only for recombinant proteins or *in vitro* reconstituted condensates is lacking (Peeples and Rosen, 2021).
- What is the actual composition and function of multilayered condensates such as ARF condensates, and how does each layer contribute to activity regulation? In humans, single-phase condensates formed by phospho-Fragile X mental retardation protein (FMRP) and CAPRIN1 homogeneously recruit RNA and the deadenylase CNOT7. By contrast, two-phase condensates formed by FMRP and phospho-CAPRIN1 concentrate RNA and CNOT7 into distinct inner and outer phases, respectively (Kim *et al.*, 2019). Despite spatial segregation, deadenylating activity was higher in the two-phase system than in the one-phase system, suggesting that enrichment does not always lead to higher activity.

## Supplementary data

The following supplementary data are available at *JXB* online.

Table S1. Examples of plant-specific condensates in various cellular compartments.

erac497_suppl_supplementary_table_S1

## Data Availability

There are no primary data associated with this manuscript.
