# Supplementary material for: Concentrating and sequestering biomolecules in condensates: impact on plant biology
Source: J Exp Bot. 2022 Dec 14;74(5):1303–8. doi: 10.1093/jxb/erac497 (PMC10010603; doi:10.1093/jxb/erac497)
Supplement: erac497_suppl_supplementary_table_S1 [file erac497_suppl_supplementary_table_s1.pdf]

Supplementary table S1

Condensates are found in all organisms, although not all form through phase separation. Prokaryotic examples of condensates are the carboxysomes, Pdu microcompartments (metabolism of 1,2-propanediol in *Salmonella enterica*), Eut bacterial microcompartments (polyhedral organelles that encapsulate major metabolic enzymes), and the RNA degradosomes (RNA metabolism in *Escherichia coli*) (Al-Husini *et al.*, 2018). There are many evolutionary conserved condensates including DNA damage bodies, stress granules, processing bodies, Cajal bodies, and nucleoli. In the table below, we provide examples of plant-specific condensates.

Name (if given)	Main proteins involved	Function(s)	Other proteins involved (if known)	Ref.
Chloroplast				
	Ankyrin-repeat proteins (STT1/2)	Intra-chloroplast cargo sorting through the chloroplast twin arginine translocation (cpTat) pathway	cpTat, OXYGEN EVOLVING COMPLEX SUBUNIT 23 KDA (OE23), HIGH CHLOROPHYLL FLUORESCENCE106 (Hcf106)	(Ouyang *et al.*, 2020)
Pyrenoid	EPYC 1 (Essential Pyrenoid Component 1)	Photosynthetic CO_2 fixation	Rubisco	(Freeman Rosenzweig *et al.*, 2017).
Nuclear				
	ABA INSENSITIVE 5 (ABI5), ABI5-INTERACTING PROTEIN 1 (AFP1)<br><br>CONSTITUTIVE PHOTOMORPHOGENESIS 1 (COP1)	Phytohormone signaling responses	ERF4	(Lopez-Molina *et al.*, 2003); (Lynch *et al.*, 2017)
	ALOG (Arabidopsis LSH1 and Oryza G1) transcription factor, TERMINATING FLOWER (TMF)	Stem cell fate transition in tomato	Floral identity protein ANANTHA	(Chen *et al.*, 2019)
	hnRNP R-LIKE PROTEIN (HRLP)	Flowering time by regulating FLC splicing and R-loop formation	Splicing factor ARGININE/SERINE-RICH 45 (SR45)	(Zhang *et al.*, 2022)
	N-methyladenosine reader Polyadenylation factor CPSF30	Floral transition and abscisic acid (ABA) response through control of polyadenylation site		(Song *et al.*, 2021)
Dicing bodies	DICER-LIKE 1 (DCL1), double-stranded RNA-binding protein 1 (DRB1), HUA ENHANCER 1 (HEN1), ARGONAUTE 1 (AGO1), SERRATE (SE)	Dicing body assembly for microRNA biogenesis, photomorphogenesis	PHYTOCHROME INTERACTING FACTOR 4 (PIF4) and other proteins	(Sun *et al.*, 2018; Xie *et al.*, 2021)
ELF	EARLY FLOWERING 3 (ELF3), GIGANTEA (GI), EARLY FLOWERING 4 (ELF4)	Thermosensing & circadian clock		(Jung *et al.*, 2020; Kim *et al.*, 2013)
Nuclear polyadenylation complex	FLOWERING CONTROL LOCUS A (FCA)	Concentrate enzymatic activity for polyadenylation (at 3′-end processing sites)	FLX-like 2 (FLL2)	(Fang *et al.*, 2019)

GBPL defence-activated condensates (GDACs)	guanylate-binding protein (GBP)-like GTPases (GBPLs); GBPL1, a pseudo-GTPase, and the catalytically active GBPL3	GDAC structures, bind defence-gene promoters and recruit specific transcriptional coactivators of the Mediator complex and RNA polymerase II for immunity	Mediator complex and RNA polymerase II	(Huang *et al.*, 2021; Kim *et al.*, 2022)
Photobodies	PHYTOCHROMES (PHYs), CRYPTOCHROMES (CRYs), UV RESISTANCE LOCUS 8 (UVR8)	Temperature response, circadian clock, photo- and thermomorphogenesis	PLASTID TRANSCRIPTIONALLY ACTIVE 12 (pTAC12 or HMR), PHOTOPERIODIC CONTROL OF HYPOCOTYL 1 (PCH1)	(Hahm *et al.*, 2020; Huang *et al.*, 2019)
Cytoplasm				
	EMBRYO DEFECTIVE 1579 (EMB1579)	Plant growth and development, *FLC* regulation, global transcription, and splicing	MULTIPLE SUPPRESSOR OF IRA 4 (MSI4), DNA Damage Binding Protein 1 (DDB1), and Cullin 4 (CUL4)	(Zhang *et al.*, 2020)
	SOSEKI proteins (DIX-dependent polymerization)-condensates accumulate at cell corners	Cellular and planar polarity	ANGUSTIFOLIA (AN)	(van Dop *et al.*, 2020; Yoshida *et al.*, 2018)
ARFs	Auxin-response factors (ARFs)	Auxin signal transduction		(Powers *et al.*, 2019)
FLOE1	FLOE1	Germination timing regulation		(Dorone *et al.*, 2021)
Hypoxia-induced SGG3 bodies	Calcium-sensor protein CALMODULIN-LIKE 38 (CML38), SUPPRESSOR OF GENE SILENCING 3 (SGG3)	Hypoxia stress, regulation of autophagy, and RNA regulatory program	AAA+-ATPase CELL DIVISION CYCLE 48A	(Field *et al.*, 2021)
Salicylic acid-induced NPR1 condensates (SINCs)	NONEXPRESSOR OF PATHOGENESIS-RELATED GENES 1 (NPR1)	Immune response effector-triggered immunity (ETI)	E3 ligase complex and various other proteins	(Zavaliev *et al.*, 2020)
siRNA bodies	SUPPRESSOR OF GENE SILENCING 3 (SGS3), RNA-DEPENDENT RNA POLYMERASE 6 (RDR6)	Transposable elements' silencing through ribosome stalling caused by unfavourable codon usage		(Kim *et al.*, 2021)

REFERENCES

Al-Husini N, Tomares DT, Bitar O, Childers WS, Schrader JM. 2018. alpha-Proteobacterial RNA Degradosomes Assemble Liquid-Liquid Phase-Separated RNP Bodies. Molecular Cell **71**, 1027-1039 e1014.

Chen F, Zhou Q, Wu L, Li F, Liu B, Zhang S, Zhang J, Bao M, Liu G. 2019. Genome-wide identification and characterization of the ALOG gene family in Petunia. BMC Plant Biology **19**, 600.

Dorone Y, Boeynaems S, Flores E, Jin B, Hateley S, Bossi F, Lazarus E, Pennington JG, Michiels E, De Decker M, Vints K, Baatsen P, Bassel GW, Otegui MS, Holehouse AS, Exposito-Alonso M, Sukenik S, Gitler AD, Rhee SY. 2021. A prion-like protein regulator of seed germination undergoes hydration-dependent phase separation. Cell **184**, 4284-4298 e4227.

Fang X, Wang L, Ishikawa R, Li Y, Fiedler M, Liu F, Calder G, Rowan B, Weigel D, Li P, Dean C. 2019. Arabidopsis FLL2 promotes liquid-liquid phase separation of polyadenylation complexes. Nature **569**, 265-269.

Field S, Conner WC, Roberts DM. 2021. Arabidopsis CALMODULIN-LIKE 38 Regulates Hypoxia-Induced Autophagy of SUPPRESSOR OF GENE SILENCING 3 Bodies. Frontiers in Plant Science **12**, 722940.

Freeman Rosenzweig ES, Xu B, Kuhn Cuellar L, Martinez-Sanchez A, Schaffer M, Strauss M, Cartwright HN, Ronceray P, Plitzko JM, Forster F, Wingreen NS, Engel BD, Mackinder LCM, Jonikas MC. 2017. The Eukaryotic CO(2)-Concentrating Organelle Is Liquid-like and Exhibits Dynamic Reorganization. Cell **171**, 148-162 e119.

Hahm J, Kim K, Qiu Y, Chen M. 2020. Increasing ambient temperature progressively disassembles Arabidopsis phytochrome B from individual photobodies with distinct thermostabilities. Nature Communications **11**, 1660.
Huang H, McLoughlin KE, Sorkin ML, Burgie ES, Bindbeutel RK, Vierstra RD, Nusinow DA. 2019. PCH1 regulates light, temperature, and circadian signaling as a structural component of phytochrome B-photobodies in Arabidopsis. Proceedings of the National Acaddemy of Sciences U S A **116**, 8603-8608.
Huang S, Zhu S, Kumar P, MacMicking JD. 2021. A phase-separated nuclear GBPL circuit controls immunity in plants. Nature **594**, 424-429.
Jung JH, Barbosa AD, Hutin S, Kumita JR, Gao M, Derwort D, Silva CS, Lai X, Pierre E, Geng F, Kim SB, Baek S, Zubieta C, Jaeger KE, Wigge PA. 2020. A prion-like domain in ELF3 functions as a thermosensor in Arabidopsis. Nature **585**, 256-260.
Kim EY, Wang L, Lei Z, Li H, Fan W, Cho J. 2021. Ribosome stalling and SGS3 phase separation prime the epigenetic silencing of transposons. Nature Plants **7**, 303-309.
Kim JH, Castroverde CDM, Huang S, Li C, Hilleary R, Seroka A, Sohrabi R, Medina-Yerena D, Huot B, Wang J, Nomura K, Marr SK, Wildermuth MC, Chen T, MacMicking JD, He SY. 2022. Increasing the resilience of plant immunity to a warming climate. Nature **607**, 339-344.
Kim Y, Lim J, Yeom M, Kim H, Kim J, Wang L, Kim WY, Somers DE, Nam HG. 2013. ELF4 regulates GIGANTEA chromatin access through subnuclear sequestration. Cell Reports **3**, 671-677.
Lopez-Molina L, Mongrand S, Kinoshita N, Chua NH. 2003. AFP is a novel negative regulator of ABA signaling that promotes ABI5 protein degradation. Genes and Development **17**, 410-418.
Lynch TJ, Erickson BJ, Miller DR, Finkelstein RR. 2017. ABI5-binding proteins (AFPs) alter transcription of ABA-induced genes via a variety of interactions with chromatin modifiers. Plant Molecular Biology **93**, 403-418.
Ouyang M, Li X, Zhang J, Feng P, Pu H, Kong L, Bai Z, Rong L, Xu X, Chi W, Wang Q, Chen F, Lu C, Shen J, Zhang L. 2020. Liquid-Liquid Phase Transition Drives Intra-chloroplast Cargo Sorting. Cell **180**, 1144-1159 e1120.
Powers SK, Holehouse AS, Korasick DA, Schreiber KH, Clark NM, Jing H, Emenecker R, Han S, Tycksen E, Hwang I, Sozzani R, Jez JM, Pappu RV, Strader LC. 2019. Nucleo-cytoplasmic Partitioning of ARF Proteins Controls Auxin Responses in Arabidopsis thaliana. Molecular Cell **76**, 177-190 e175.
Song P, Yang J, Wang C, Lu Q, Shi L, Tayier S, Jia G. 2021. Arabidopsis N(6)-methyladenosine reader CPSF30-L recognizes FUE signals to control polyadenylation site choice in liquid-like nuclear bodies. Molecular Plant **14**, 571-587.
Sun Z, Li M, Zhou Y, Guo T, Liu Y, Zhang H, Fang Y. 2018. Coordinated regulation of Arabidopsis microRNA biogenesis and red light signaling through Dicer-like 1 and phytochrome-interacting factor 4. PLoS Genetics **14**, e1007247.
van Dop M, Fiedler M, Mutte S, de Keijzer J, Olijslager L, Albrecht C, Liao CY, Janson ME, Bienz M, Weijers D. 2020. DIX Domain Polymerization Drives Assembly of Plant Cell Polarity Complexes. Cell **180**, 427-439 e412.
Xie D, Chen M, Niu J, Wang L, Li Y, Fang X, Li P, Qi Y. 2021. Phase separation of SERRATE drives dicing body assembly and promotes miRNA processing in Arabidopsis. Nature Cell Biology **23**, 32-39.
Yoshida S, van der Schuren A, van Dop M, van Galen L, Saiga S, Adibi M, Moller B, Marhavy P, Smith R, Friml J, Weijers D. 2018. A SOSEKI-based coordinate system interprets global polarity cues in Arabidopsis. 479113.
Zavaliev R, Mohan R, Chen T, Dong X. 2020. Formation of NPR1 Condensates Promotes Cell Survival during the Plant Immune Response. Cell **182**, 1093-1108 e1018.
Zhang Y, Fan S, Hua C, Teo ZWN, Kiang JX, Shen L, Yu H. 2022. Phase separation of HRLP regulates flowering time in Arabidopsis. Science Advances **8**, eabn5488.
Zhang Y, Li Z, Chen N, Huang Y, Huang S. 2020. Phase separation of Arabidopsis EMB1579 controls transcription, mRNA splicing, and development. PLoS Biology **18**, e3000782.
